# Reviewed article: Luis MA, Mota JC, D’Oliveira CAFB, Vasconcelos CH, Silva RP, Junior ABD, Regina FL, Lima CM, Sá NB, Cardoso LO. Factors associated with transgender people mortality due to violent causes: a cross-sectional study, Brazil, 2014-2022. Epidemiol Serv Saude. 2026:35;e20240748

**DOI:** 10.1590/S2237-96222026v35e20240748.b

**Published:** 2026-03-16

**Authors:** Guilherme Lamperti Thomazi

**Affiliations:** 1 Aids Healthcare Foundation, Porto Alegre, RS, Brazil

## Peer review B

## Questionnaire

Does the manuscript contain new and significant information to justify publication? Yes

Does the Abstract (Summary) clearly and accurately describe the content of the article? Yes

Is the problem significant and concisely stated? No

Are the methods described comprehensively? Yes

Are the interpretations and conclusions justified by the results? No

Is adequate reference made to other work in the field? Yes

## Manuscript Structure

Length of article is: Adequate

Number of tables is: Adequate

Number of figures is: Too few

Please state any conflict(s) of interest that you have in relation to the review of this paper. None

Interest: Excellent

Quality: Average

Originality: Excellent

Overall: Good

Recommendation: Minor Revision

## Comments

Parabens pelo artigo que traz dados extremamente valiosos e ainda tão pouco trabalhados sobre essa população. Faço alguns comentários, como sugestões, no corpo do texto.

Minha maior questão é em relação a organização da discussão, parece que ela “vai e volta” muitas vezes, fazendo com que a pessoa leitora fique um pouco perdida.

Fala de causas violentas, depois muda de assunto e depois volta à violencia sexual.

Os dados quantitativos sobre pessoas trans são bastante incipientes, sugiro comparação com a população em geral como na parte em que falam de suicidio e mesmo nas causas de morte, mostrando as especificidades entre pessoas trans e a população em geral (como idade). Além disso, comparar com a população negra e jovem que também tem altissimas mortalidade por arma de fogo.

Seria interessante também um maior contato com as políticas (ou falta delas) em relação a população estudada. Fala, por exemplo, de que a notificação possa ter aumentado por causa de possiveis capacitações mas passamos por 4 anos de desmonte de políticas voltadas a essa população.

Como frisado no artigo, ele aborda dados inéditos. Sugiro colocar que grande parte dos dados discutidos foram levantados por ONGs ou pesquisas com poucos individuos, e que é a primeira utilizando um banco de dados tão grande como o SINAM.

Senti falta em uma discussão um pouco mais abrangente sobre o suicidio de homens trans, como alertado por movimentos sociais.

Sugiro padronizar nomenclaturas em relação a pessoas trans: Mulher trans e travesti, Homem trans. Como usado pelo movimento social. Sugiro tambem uma breve explicação sobre quem são essas pessoas.

Como falei no início, parabéns pelo artigo e esses dados serão fundamentais para trabalhos futuros bem como políticas públicas de diferentes áreas.

